# Supplementary material for: DNA mechanocapsules for programmable piconewton responsive drug delivery
Source: Nat Commun. 2024 Jan 24;15:704. doi: 10.1038/s41467-023-44061-w (PMC10808132; doi:10.1038/s41467-023-44061-w)
Supplement: Supplementary file 1 — Supplementary information [file 41467_2023_44061_MOESM1_ESM.pdf]

Supplementary Information

DNA mechanocapsules for programmable piconewton responsive drug delivery

Arventh Velusamy[1], Radhika Sharma[1], Sk Aysha Rashid[1], Hiroaki Ogasawara[1] and Khalid Salaita[1,2]

[1]Department of Chemistry, Emory University, Atlanta, GA, USA.
[2]Wallace H. Coulter Department of Biomedical Engineering, Georgia Institute of Technology and Emory University, Atlanta, GA, USA
E-mail: k.salaita@emory.edu

TABLE OF CONTENTS

Supplementary Note 01: DNA mechanocapsules for precision biophysical targeting *in vivo*

Supplementary Table 01: Comparison of force-triggered drug delivery systems reported in literature
Supplementary Table 02: Primer sequences used for RT-qPCR
Supplementary Table 03: Oligonucleotides and chemical modifications used for DMCs

Supplementary Figure 02: DNA mechanocapsule design and orientation of function groups
Supplementary Figure 03: Tuning force-thresholds of chemically identical DMCs
Supplementary Figure 04: Engineering DMCs that are non-responsive to cellular forces
Supplementary Figure 05: Rupture dynamics of DMC is independent of force orientation
Supplementary Figure 06: Fluorophore dequenches on DMC rupture regardless of force direction
Supplementary Figure 07: Characterization of DMC formation
Supplementary Figure 08: Surface density of DMCs
Supplementary Figure 09: Encapsulation of Dextran inside DMCs
Supplementary Figure 10: Differential cargo uptake in a co-culture of high and low force MEF cells
Supplementary Figure 11: Quantifying ASO dose-response function
Supplementary Figure 12: Flow Cytometry gating strategy
Supplementary Figure 13: Uncropped gel images

Supplementary Note 01: DNA mechanocapsules for precision biophysical targeting *in vivo*

DNA mechanocapsules (DMCs) offer tunable, well-defined force thresholds for targeting and precision drug-delivery to cells with elevated levels of receptor forces. DMCs can improve the specificity of cargoes by utilizing biophysical phenotypes for drug release in addition to the chemical selectivity of ligand-receptor binding.

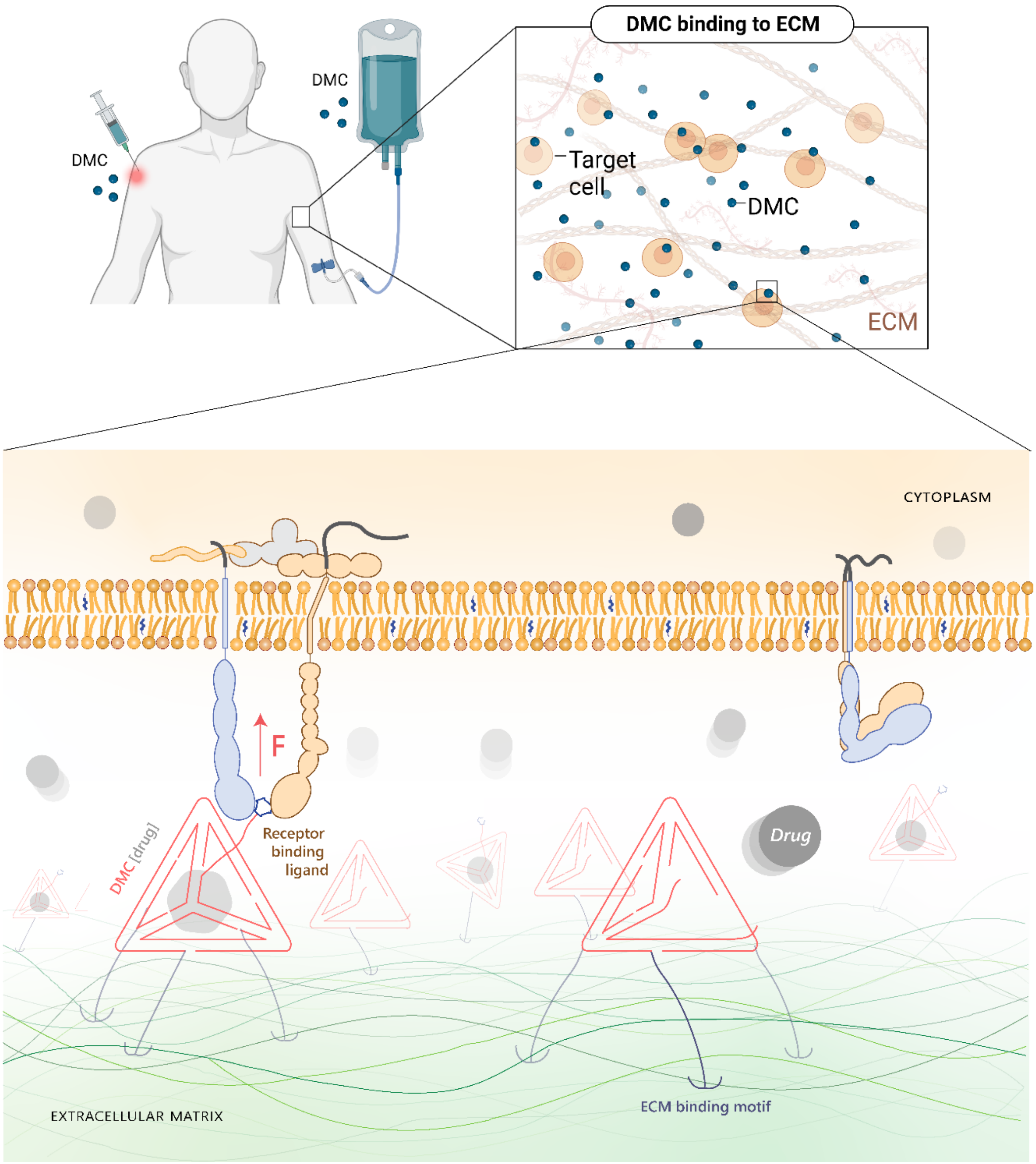

Supplementary Figure 01: DNA mechanocapsules (DMCs) offer precision drug-delivery to diseased tissues by targeting mechanical phenotypes.

In principle, DMCs bridging any two macromolecular complexes can be pulled open by piconewton forces to release cargo. Hence, a third layer of selectivity can be stacked upon the current design in an *in vivo* context by simply replacing the methyltetrazine anchor with a tissue specific anchor. For example, DMCs could be tethered to ECM using homing ligands such as such as fibronectin binding peptides and ECM-specific antibodies[2–4]. The DMCs' design allows for arbitrarily increasing the anchoring strength that can withstand the molecular forces by decorating the DMC with multiple binders.

Furthermore, a localized force-based release mechanism benefits from the ability to produce a high concentration of drug in intimate proximity to the cell of interest. For example, only 1000 drug molecules are required to achieve a concentration of 1 nM in a HeLa cell. Hence, DMCs can protect a broad-spectrum drug safely until it reaches its destination and release it there for maximum efficacy. This vastly reduces the concentration of therapeutics required thereby minimizing off-target effects of systemic delivery.

Supplementary Table 01: Comparison of force-triggered drug delivery systems reported in literature

The vast majority of drug delivery systems use biochemical cues but a few did use biophysical cues such as forces. The table below lists various systems that have been developed to release cargo based on biophysical cues. The first example responds to bulk shear forces and is not sensitive to molecular forces. The second example can only be used to deliver drugs that bind to a specific aptamer and hence the impact is narrow. The third nanoporous silica example employ micron sized beads and hence it is not likely suitable for *in vivo* drug delivery. DMCs are modular both in terms of magnitudes of forces and the classes of encapsulated cargo.

Mechanosensitive platform	Force sensitivity	Tunable	Drug cargo	Vehicle size	Journal (Year)
Polymer aggregates	N/A (Shear-induced)	No	Protein	~1-5 μm	*Science* (2012)[1]
DNA aptamers	N/A	No	Only 1 type of protein specific to aptamer	~ 10 nm	*Advanced Materials* (2019)[2]
Nanoporous silica microparticles	~50 pN	No	Small molecule	~1-5 μm	*Materials Horizons* (2020)[3]
DNA Mechano Capsules (DMC)	27 – 44 pN	Yes	Macromolecules, DNA/RNA	~10 nm	Current work

Supplementary Table 02: Primer sequences used for RT-qPCR

Primer sequences used for used for RT-qPCR. DNA strands were custom-synthesized by Integrated DNA Technologies (Coralville, IA, USA).

Name	Sequence
HIF1 α – forward primer	TAT GAG CCA GAA GAA CTT TTA GGC
HIF1 α – reverse primer	CAC CTC TTT TGG CAA GCA TCC TG
18S – forward primer	AGG AAT TGA CGG AAG GGC ACC A
18S – reverse primer	GTG CAG CCC CGG ACA TCT AAG

Supplementary Table 03: Oligonucleotides and chemical modifications used for DMCs

DNA strands were synthesized by Integrated DNA Technologies. The modifications in the oligonucleotides are highlighted depending on the chemistry used for linking the functional group as follows: 1) Alkyne-azide copper click 2) SMCC-thiol 3) Amine NHS-ester 4) commercially available modification. The retention times of the modified strands are also provided wherever applicable.

Name	R_t (min)	Sequence
TD1-S1a	—	CCAGGCAGTTGAGACGAACAT
TD1-S1b	—	TCCTAAGTCTGAAATTTATCACCCGCCATAGTAGACGTATCA
TD1-S2	—	AGCTTGCTACACGATTCAGACTTAGGAATGTTCGA<br>CATGCGAGGGTCCAATACCGACGATTAC
cRGD_TD1-S3a_BHQ	23.0	/cRGD/ GTGATAAAACGTGTAGCAAGCTGTAATCGACGGGAAG /BHQ2/
Cy3B_TD1-S3b	17.7	/Cy3B/ AGCATGCCCATCCACTACTATGGCGG
Tz_TD1-S4	13.7	/Tetrazine/ CTCGCATGACTCAACTGCCTGGTGATACGAGGATG<br>GGCATGCTCTTCCCGACGGTATTGGACC
Tz_TD3-S4_cRGD	14.4	/Tetrazine/ CTCGCATGACTCAAC /cRGD/<br>GCCTGGTGATACGAGGATGGGCATGCTCTTCCCGACGGTATTGGACC
S3a Invader	—	CTTCCCGTCGATTACAGCTTGCTACACGTTTTATCA CTCTCTCTC
S3a toe BHQ	—	GAGAGAGA GTGATAAAACGTGTAGCAAGCTGTAATCGACGGGAAG /BHQ2/
S3a toehold	—	GAGAGAGA GTGATAAAACGTGTAGCAAGCTGTAATCGACGGGAAG
TD4-S1	—	CCAGGCAGTTGAGACGAACATTCCTAAGTCTGAAATTT<br>ATCACCCGCCATAGTAGACGTATCA
TD4-S3	—	GTGATAAAACGTGTAGCAAGCTGTAATCGACGGGAAG<br>AGCATGCCCATCCACTACTATGGCGG
cRGD_TD5-S3a_A647	16.3	/cRGD/ GTGATAAAACGTGTAGCAAGCTGTAATCGACGGGAAG /Alexa647/
cRGD_TD6-S3a_HIF1a	15.0	/cRGD/ GTGATAAAACGTGTAGCAAGCTGTAATCGACGGGAAG /Thiol/<br>+T*+G*+G*C*A*A*G*C*A*T*C*C*+T*+G*+T*A /SMCC/
TD7-S2	—	TAGGAATGTTCGACATGCGAGGGTCCAATACCGA<br>CGATTACAGCTAGCTACACG A TTCAGACT
cRGD_TD7-S3a BHQ	21.6	/cRGD/ GTGATAAAACGTGTAGCTA /BHQ2/
cRGD_TD7-S3a	15.0	/cRGD/ GTGATAAAACGTGTAGC /T-NH$_2$/ A
Cy3B_TD7-S3b	17.0	/Cy3B/ GCTGTAATCGACGGGAAGAGCATGCCCATCCACTACTATGGCGG
cRGD_TD8-S3a	14.9	GTGATAAAACGTG /cRGD/ AGCT /T-NH$_2$/ A
cRGD_TD8-S3a_BHQ	21.3	GTGATAAAACGTG /cRGD/ AGCTA /BHQ2/
TD8-S3a no mod	—	GTGATAAA A CGTGTAGCTA
HIF1a_SMCC	19.0	+T*+G*+G*C*A*A*G*C*A*T*C*C*+T*+G*+T*A /SMCC/
HIF1a_SH	—	+T*+G*+G*C*A*A*G*C*A*T*C*C*+T*+G*+T*A /Thiol/
HIF1a_TD1-S3b	15.9	+T*+G*+G*C*A*A*G*C*A*T*C*C*+T*+G*+T*A /Thiol/—/SMCC/ AGCATGCCCA<br>TCCACTACTATGGCGG
TD5-S3a_A647	14.7	GTGATAAAACGTGTAGCAAGCTGTAATCGACGGGAAG /Alexa647/
cRGD_TD1-S3a	15.2	/cRGD/ GTGATAAAACGTGTAGCAAGCTGTAATCGACGGGAAG
cRGD_TD6-S3a_(HIF1a)$_2$	18.0	/cRGD/ GTGATAAAACGTGTAGCAAGCTGTAATCGACGGGAAG /dithiol/<br>**2x** {+T*+G*+G*C*A*A*G*C*A*T*C*C*+T*+G*+T*A /SMCC/}

Supplementary Table 03 (continued): Oligonucleotide modifications

DNA strand modifications used for the modification of DMCs. Cy3B-NHS ester, SMCC, Alexa 647-NHS ester and Tetrazine-NHS ester were used to modify amine terminus or T-nucleobase amine on DNA strands. Tetrazine-N_3 and cRGD-N_3 were used to modify alkyne terminus or alkyne on T- nucleobase. Other modifications are obtained commercially from Integrated DNA Technologies.

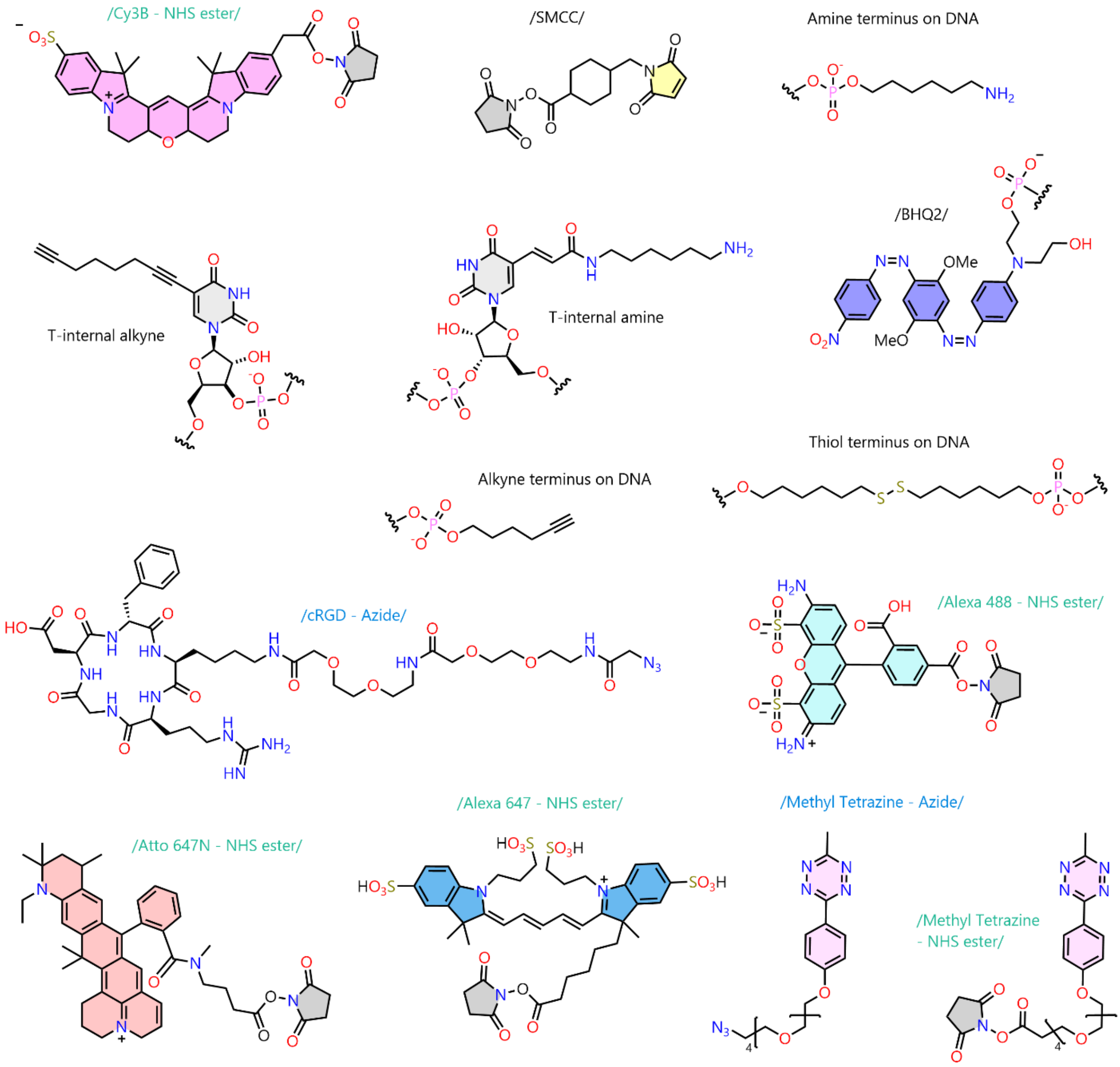

Supplementary Figure 02: DNA mechanocapsule design and orientation of functional groups

Tetrahedron (20 bp edge) reported in from A. J. Turberfield et al. 2005[4] was modified to have 6 nicks. (**a**) Complementary regions of the tetrahedron DNA are colors matched and unpaired A hinges are colored turquoise. (**b**) The nicks on the tetrahedron can be chemically modified to provide attachment points. Fluorophores, quenchers, and drug cargoes are placed on the 8th base to shield them inside the tetrahedron while RGD ligands and tetrazine anchors are placed on the 13th base to display on the outside the tetrahedron. (**c**) The attachment point is on the inside (gray) of the tetrahedron for a conjugation at nucleotide 8 and on the outside (dark blue) of the tetrahedron at nucleotide 13. Base counting is done in the 5′ to 3′ direction, starting with the unpaired nucleotide at the hinge as zero.

a

```
                           0         1         2
                         - 12345678901234567890 -
S1a   CCAGGCAGTTGAG A CGAACAT
S1b                              TCCTAAGTCTGAA A TTTATCACCCGCCATAGTAG A CGTATCA
S2    AGCTTGCTACACG A TTCAGACTTAGGAATGTTCG A CATGCGAGGGTCCAATACCG A CGATTAC
S3a    cRGD GTGATAAA A CGTGTAGCAAGCTGTAATCG A CGGGAAG BHQ2
S3b                                            Cy3B AGCATGCCCATCC A CTACTATGGCGG
S4   Anchor CTCGCATG A CTCAACTGCCTGGTGATACG A GGATGGGCATGCTCTTCCCG A CGGTATTGGACC
                                                0         1         2
                                              - 12345678901234567890 -
```

b

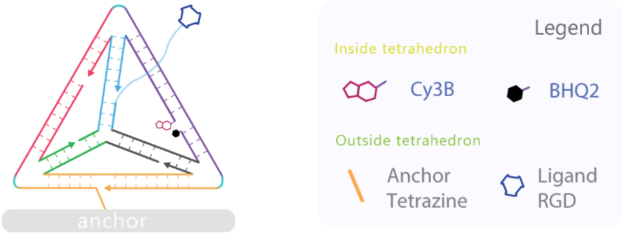

c

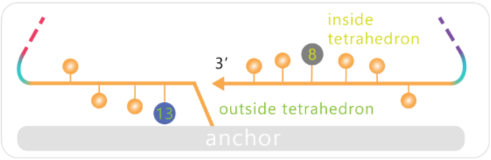

Supplementary Figure 03: Tuning force-thresholds of chemically identical DMCs

Force threshold of DMCs were tuned by decreasing the length of force-bearing strand in DMC_{39pN}. Further, the RGD ligand was linked to the middle of the force-bearing strand in another DMC. The RGD attachment points were pulled along the z-axis at a loading rate of 1.41×10^4 nm/s. Coarse-grain modeling of the DMCs were performed using a sequence independent oxDNA2 model. (**a, b**) The force-bearing strand sequence can be chemically modified using internal alkynes (facing outward, blue M) and amines (facing inward, gray M) for the attachment of ligand and cargo respectively. (**c,d**) The DMCs were modelled using oxDNA and their force extension curves were plotted. It is plotted along with their exponential moving averages of 40 data points (**e, f**) The number of base pairs in the DMC as a function of time were also extracted from the simulation and plotted below. (**g**) The DMC_{29pN} was pulled at the rate of 1.4×10^6 nm/s (red), 1.4×10^5 nm/s (blue), 1.4×10^4 nm/s (yellow) along the z-axis in oxDNA and the data was smoothened using an exponential smoothening function. (h) Shearing force estimate of 21 bp dsDNA with an identical loading rate of 1.4×10^4 nm/s.

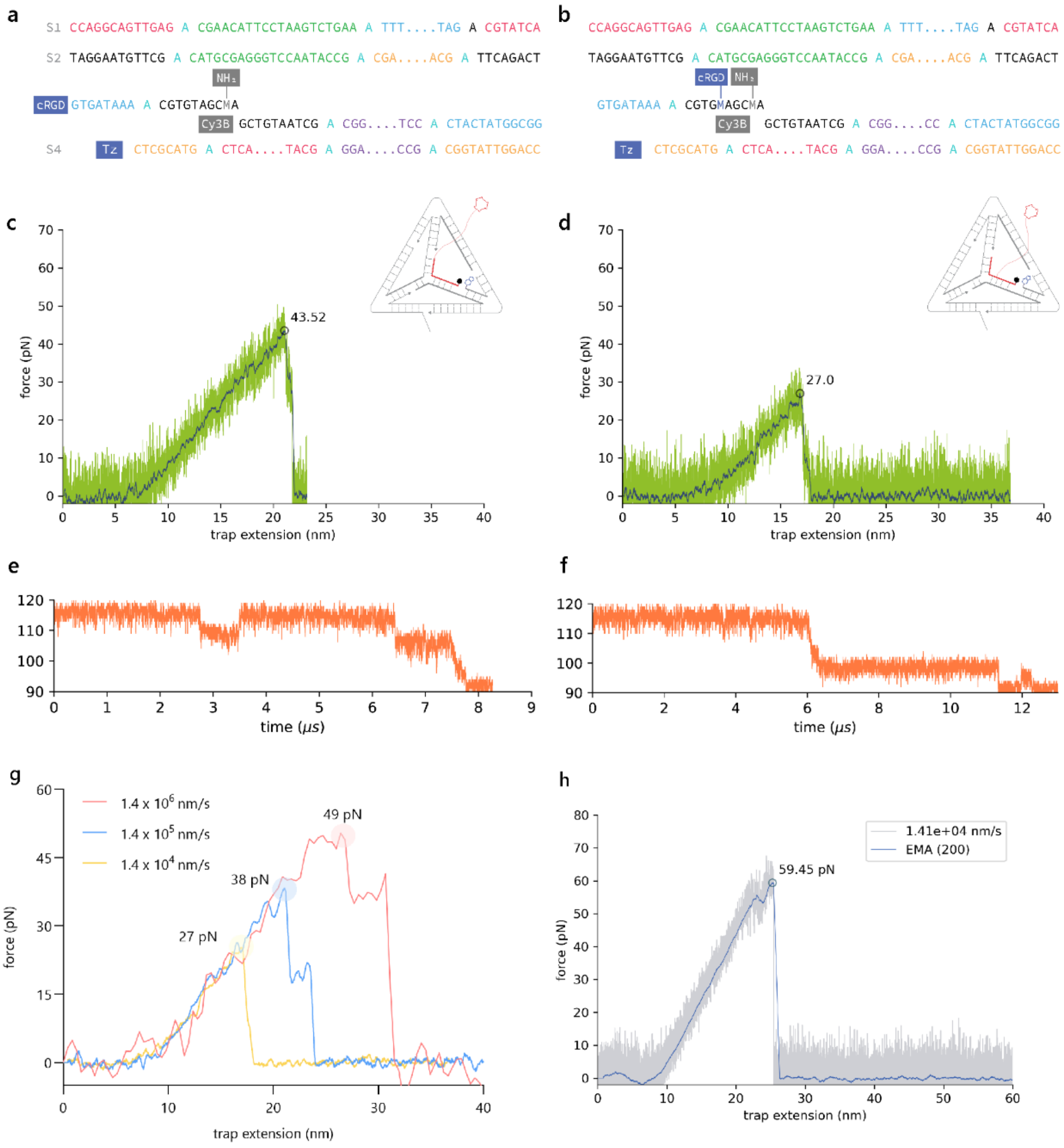

Supplementary Figure 04: Engineering DMCs that are non-responsive to cellular forces

DMCs (with force pulling positions 1-5) were subjected to force ramps with peak force as high as 500 pN in oxDNA and the number of base pairs ruptured due to the force were recorded as a function of time. These specific bases were chosen since only T bases can be modified with alkyne handles commercially and among the T bases only those that had the modifications displayed on the outside were selected. (**a**) Different points along the anchor strand on which the force was applied while the anchoring strand was immobilized. The values in circles represent the percentage of total base pairs ruptured at the end of a simulation. (**b**) Change in base pairs due to increasing forces as a function of time in nanoseconds. The nucleotides on the anchoring strands (1-5) were pulled along the z-axis at a loading rate of 2.81×10^5 nm/s. (**c, d**) The separation distance between the Cy3B and BHQ2 attachment points on the DMCs as a function of time was measured to check for Cy3B dequenching. In the case of DMC-1 (DMC_{rigid}) the fluorophore-quencher pairs are maintained within FRET radius. On the contrary, DMC-5 had well separated fluorophore-quencher pairs by the end of simulation indicating structural collapse.

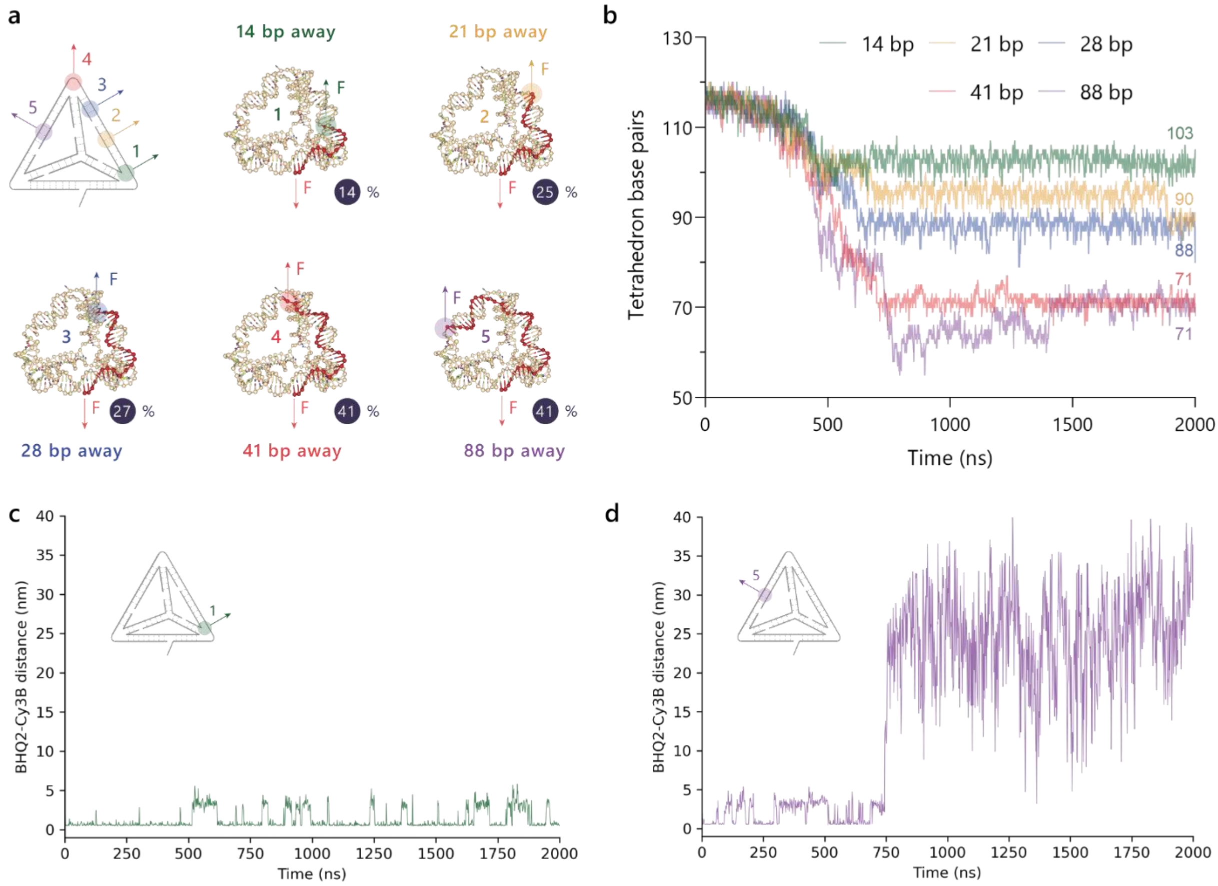

Supplementary Figure 05: Rupture dynamics of DMC is independent of force orientation

The influence of force orientation on the threshold and rupture dynamics of DMC_{39pN} was tested with forces vectors along different directions. (**a, c, e, g, i, k**) Force extension curves of DMC_{39pN} shows the peak force of rupture for a specific force orientation in oxDNA simulation. (**b, d, f, h, j, l**). Distance of separation of strands from its corresponding complement in the DMC as the force ramps up.

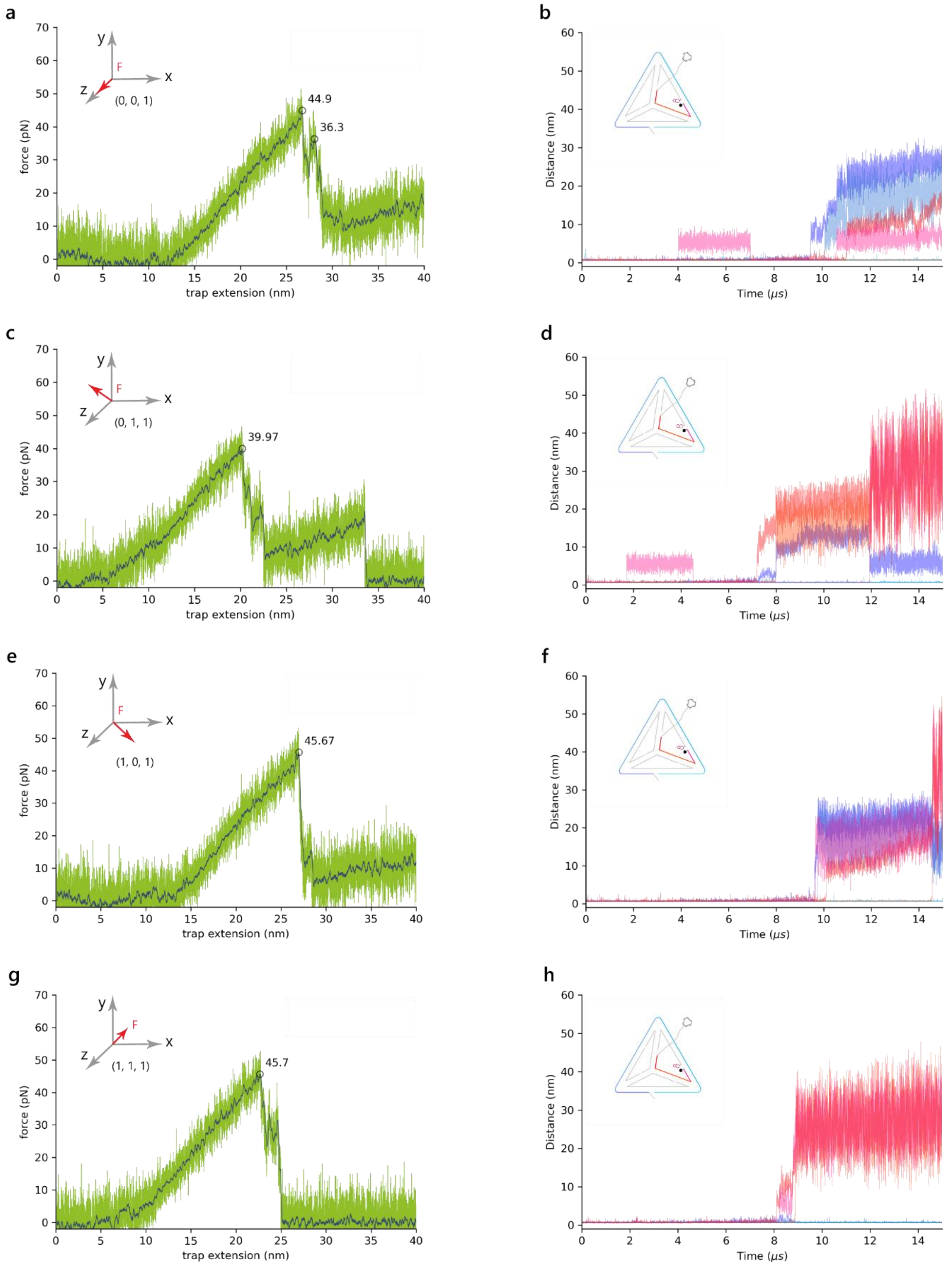

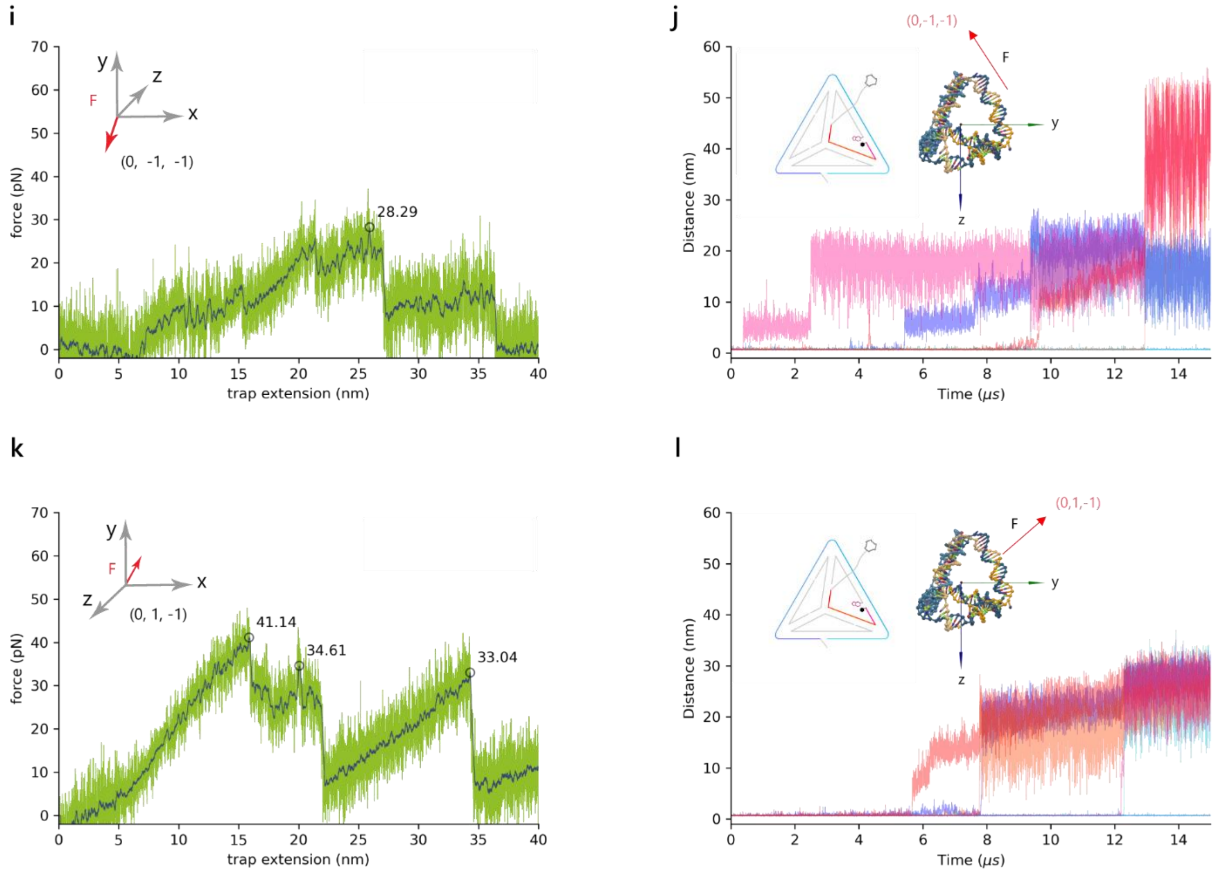

In most cases, the force of rupture fluctuates within a few piconewtons range due to the inherent stochasticity of coarse grain simulations. It must be noted that the force orientation of (0, -1, -1) would be physically impossible due to the steric hindrance of integrin-ligand complexes. Since the size of the integrin is much larger than the size of the DMC, it would be impossible to exert forces along the axis of the ligand modified edge.

Supplementary Figure 06: Fluorophore dequenches on DMC rupture regardless of force direction

DMC_{39pN} was tested with forces along different orientations and the number of base pairs as well as the fluorophore-quencher distances were extracted from the simulation. The number of base pairs in the DMC (grey) and the quencher BHQ2, fluorophore Cy3B separation (orange) on the DMC are plotted for forces in the direction of (**a**) z axis (**b**) y, z axis (**c**) x, z axis (**d**) x, y, z axis (**e**) -y, -z axis (**f**) y, -z axis.

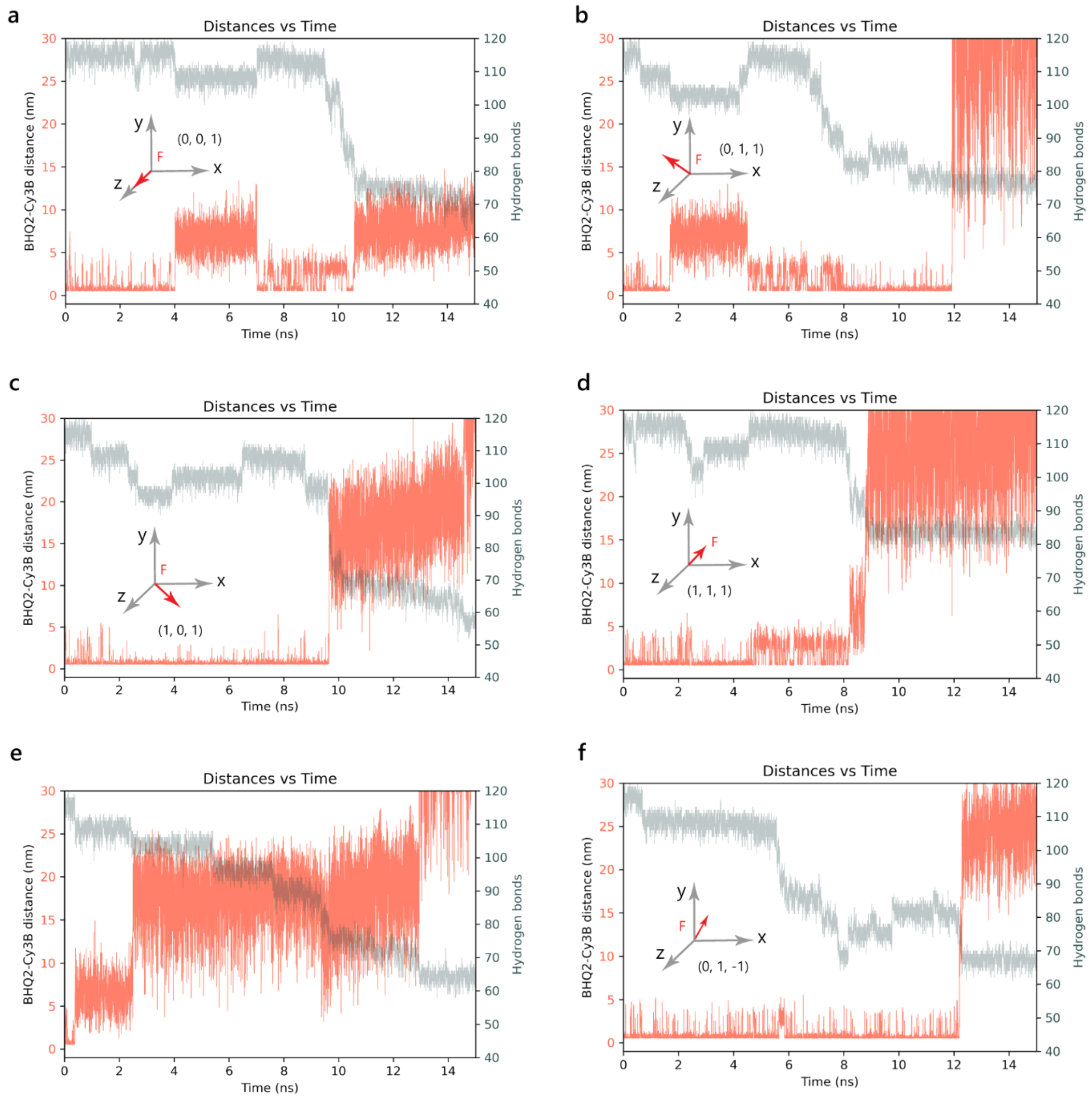

In all cases, the BHQ2-Cy3B pairs remain well separated beyond the 10 nm FRET radius thereby leading to an increase in fluorescence when the force-bearing strand is ruptured from the DMC.

Supplementary Figure 07: Characterization of DMC formation

(**a**) DMC_{39pN} strands (without chemical modifications) of varying combinations at 1 μM were subjected to the DMC annealing protocol and were run using 3.5% agarose gel at 100 V. **Lane 9:** shows DMC formation along with higher-order structures due to elevated concentration. (**b**) DMC with various chemical modifications at 50 nM concentrations. DMCs fold with high yield (>95%) when annealed at lower concentrations. (**c**) DNA tetrahedron with 4 strands (lane 6) reported by Goodman et al.[4] along with the DMC (lane 5) and the individual tetrahedron strands for comparison (lane 1 - 4). (**d**) Dynamic light scattering of the DMC recorded at 25°C.

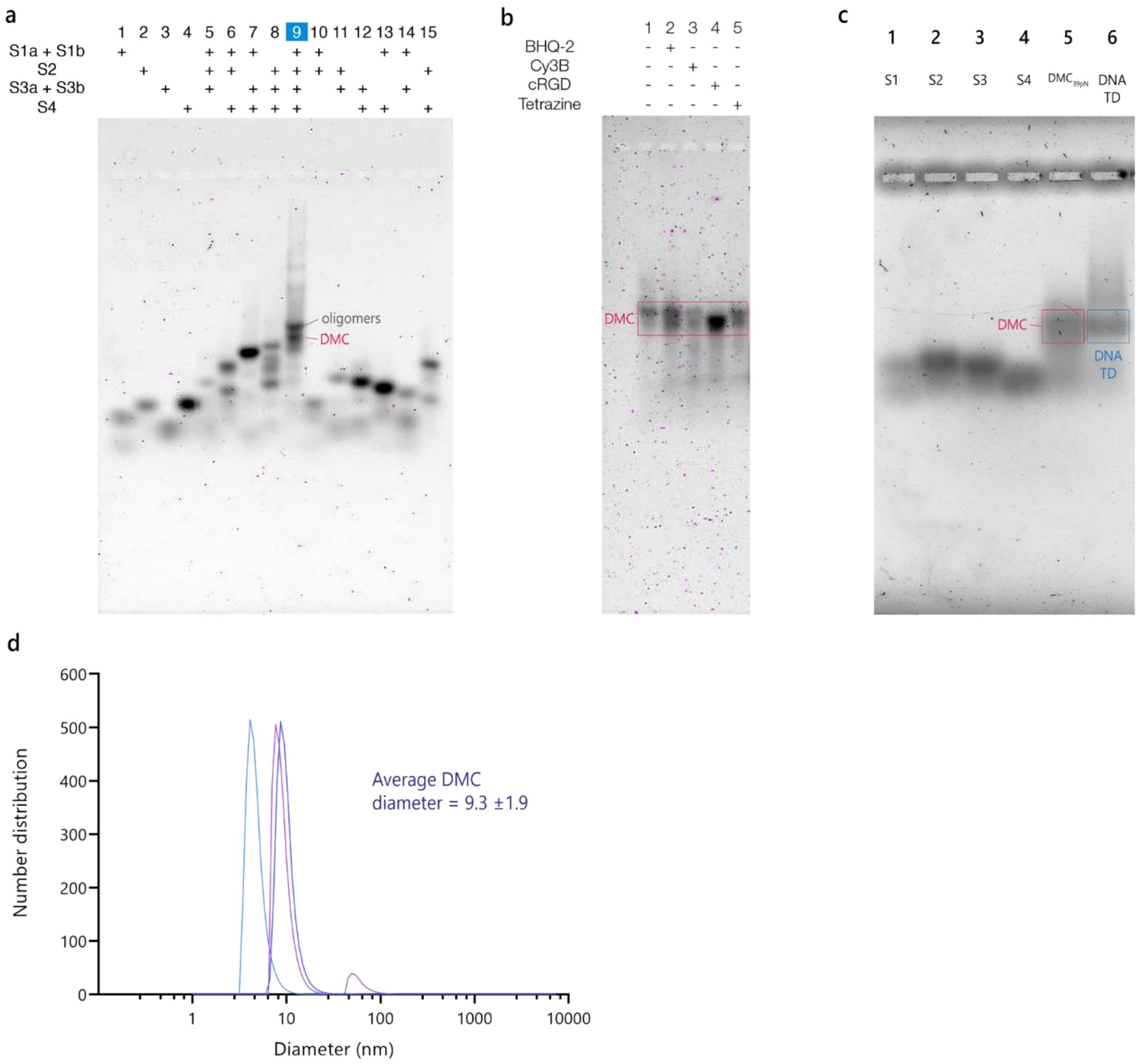

Supplementary Figure 08: Surface density of TDs

DMCs with Cy3B of various concentrations were clicked to the surfaces for 1 hour and the intensity on the surface after washing the unbound DMCs were measured. (**a, b**) Schematic of increasing DMC concentration immobilized on the surface with increasing DMC solution concentration. The intensity of Cy3B labelled DMC on the surface was transformed to the number of DMCs/μm^2 using Supported Lipid Bilayer (SLB) calibration curve. (**c**) The DMC density was converted to average distance between two DMCs on the surface. The 50 nM has a 3.72 standard error of mean. (**d**) SLB calibration: Intensity of Texas Red DHPE (N-(Texas Red sulfonyl)-1,2-dihexadecanoyl-snglycero-3-phosphoethanolamine, triethylammonium salt) to the number of molecules on the surface. (**e**) F-factor plot estimation using concentrations of labelled oligonucleotide and SUVs (small unilamellar vesicles) in solution to compare the fluorescence intensity with density. The ratio of the calibration curve slopes was used to determine the "F factor" for the labelled oligonucleotide and the SUV samples. The F-factor was calculated as follows: F=$I_{\mathrm{Cy3B}-DNA}/I_{\mathrm{TR}-DHPE}$, where $I_{\mathrm{Cy3B}-DNA}$ and $I_{TR\text{-}DHPE}$ represent the fluorescence intensities of the DNA and SUV samples, respectively.

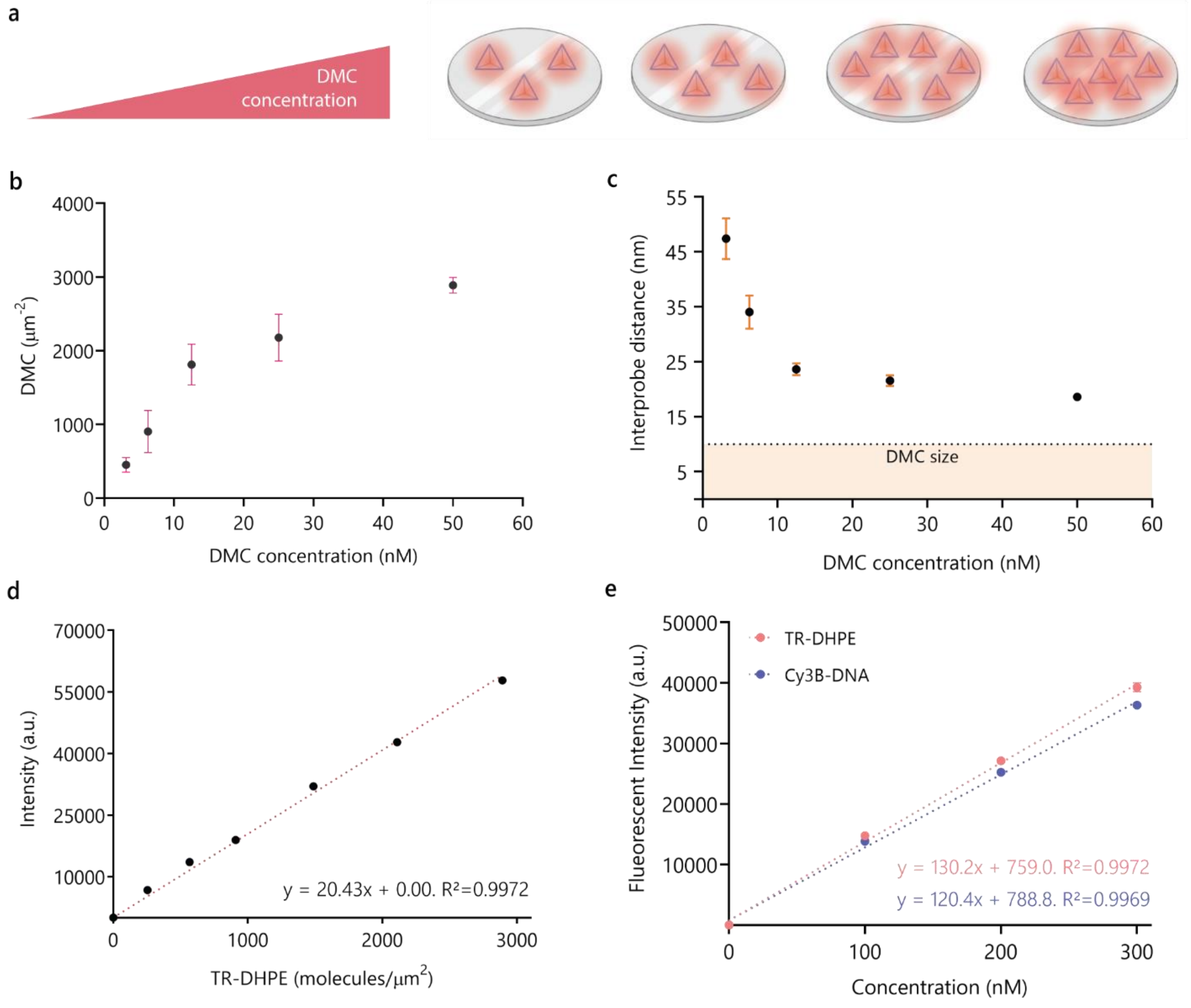

Supplementary Figure 09: Encapsulation efficiency of dextran inside DMCs

(**a**) Assembly and purification protocol for the dextran encapsulated DMCs. (**b**) Validating Cy5-labelled dextran (Dex_{Cy5}) encapsulation in DMCs. **Lane 1:** DMC. **Lane 2:** Dex_{Cy5}. **Lane 3:** DMC and Dex_{Cy5} annealed (Δ) together **Lane 4:** DMC and Dex_{Cy5} annealed separately and mixed **Lane 5:** DMC and Dex_{Cy5} annealed together at 10 μM and purified (◊) using 30 kDa filters. **Lane 6:** DMC and Dex_{Cy5} annealed together at 100 μM and purified (**c**) Toehold mediated release of Dex_{Cy3B} from DMC with BHQ2 (orange dots), DMC with Cy3B and BHQ2 without encapsulated dextran (brown dots, positive control), Cy3B release from DMC with BHQ2 in the presence of a scrambled invader (beige dots, negative control) (**d**) Agarose gel (3.5%) ran with DMC and varying concentrations of Dex_{Cy5} (Standard concentration - 200 nM Dex_{Cy5}). Encapsulation (%) = 100 x $\frac{I_{well}}{I_{standard}}$, where I is the integrated intensity of the lane after background subtraction. Size exclusion chromatograph of (**e**) Dex_{Cy5} (**f**) DMC.

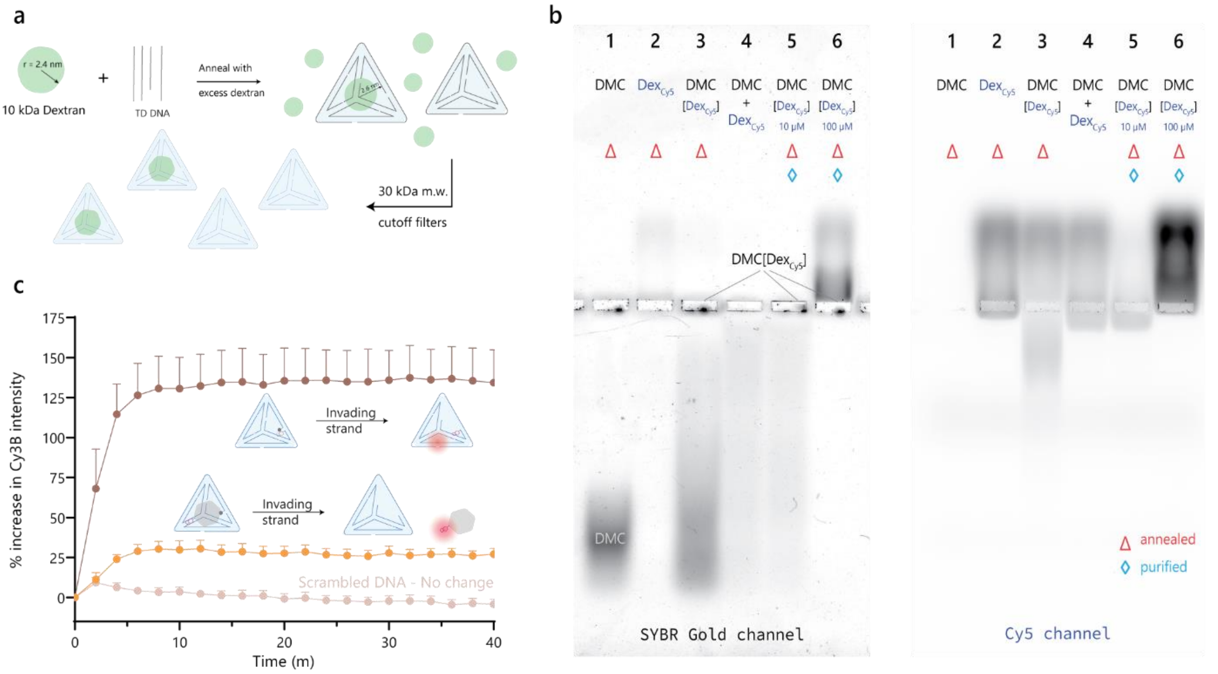

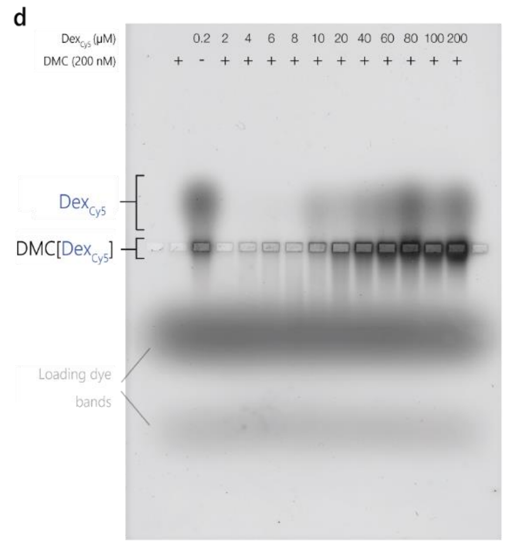

Dex_{Cy5} (μM)	Encapsulation %	Normalized lane intensity
0.2 (Std)	-	100
2	0	63
4	0	59
6	2	61
8	2	62
10	7	74
20	14	83
40	25	99
60	31	114
80	43	137
100	35	116
200	44	126

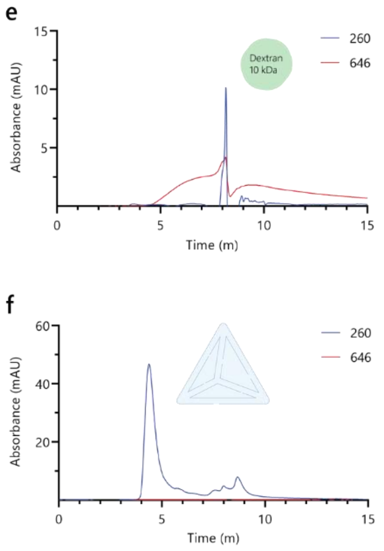

Supplementary Figure 10: Differential cargo uptake in a co-culture of high and low force MEF cells

MEF cells were seeded on DMC_{39pN} $[Dex_{647N}]$ functionalized surfaces and the uptake was measured using flow cytometer after 2 hrs. Vinculin (GFP) vs Dextran uptake (Atto647N) plot of (**a**) MEF cells expressing GFP-tagged Vinculin (GFP-Vin), (**b**) MEF cells with Vinculin knocked out (VinKO), (**c**) an overlay of (**a**,**b**), (**d**) a co-culture of MEF GFP-Vin and MEF VinKO on the same DMC_{39pN} $[Dex_{647N}]$ surfaces, (**e**) normalized uptake of Vin-GFP compared to the VinKO cells cultured on separate surfaces.

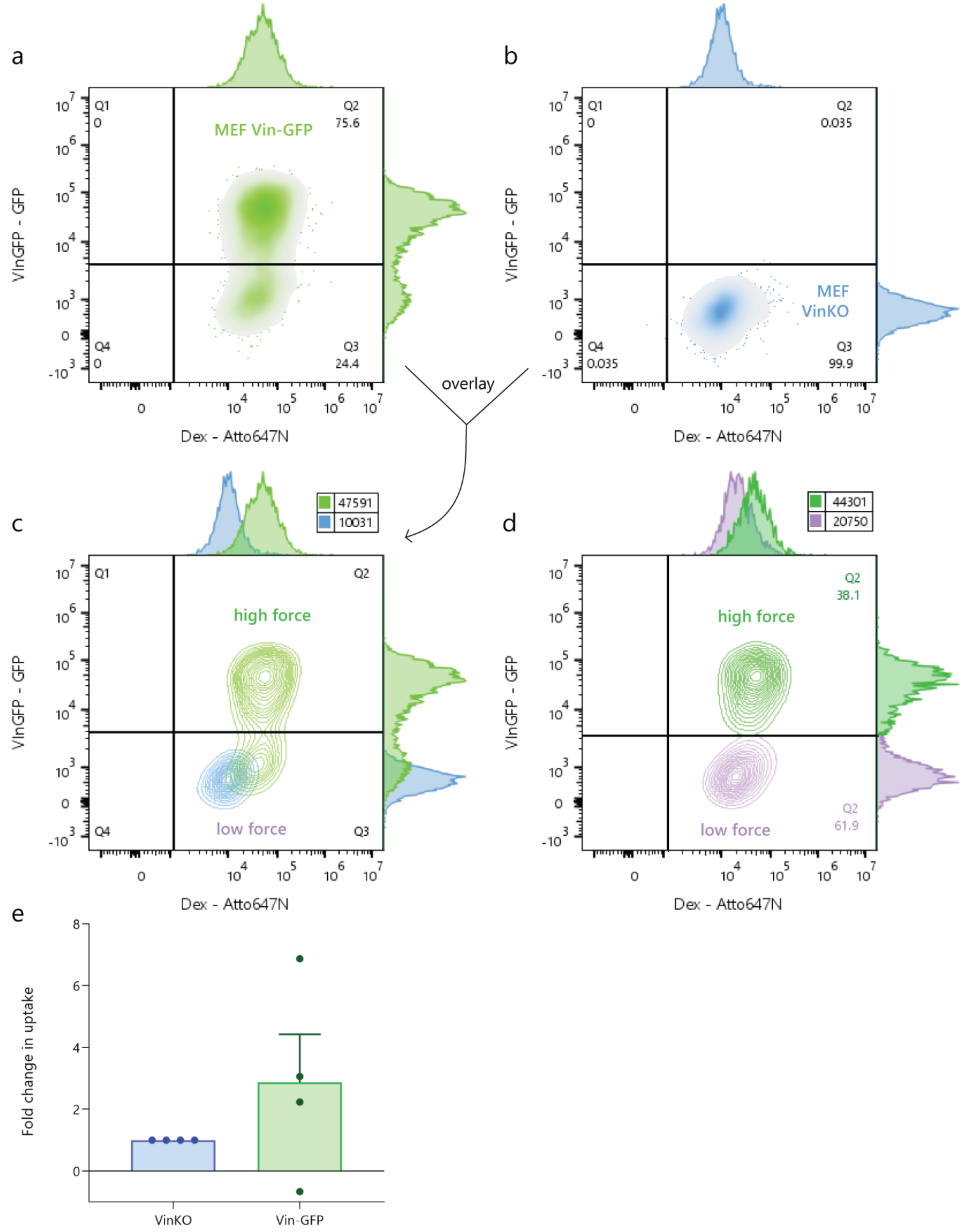

Supplementary Figure 11: Quantifying ASO dose-response function

HeLa cells were treated with HIF-1α antisense oligonucleotide, and the mRNA knockdown was quantified using qRT-PCR. Briefly cells were seeded in 10% FBS in DMEM for 24 hours in 12 well plates and then the media was replaced with 500 µl of optiMEM media containing different concentrations of the antisense drug. After 4h the FBS was added to the media and the cells were allowed to grow for another 20 hrs. At the end of 2nd day, cells were lysed, and the mRNA levels were analyzed using RT-qPCR. Each dot represents a biological replicate, and the error bars denote SEM. HIF-1α mRNA levels were measured using 18S as a reference gene. The numbers in the bar graph denote the reduction in mRNA expression levels.

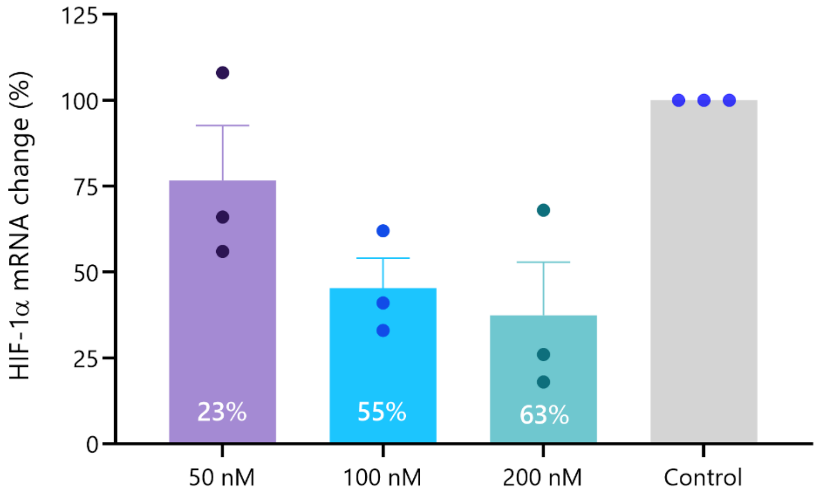

To achieve a mRNA knockdown of 23%, a concentration of 50 nM of the drug in 500 µL of the media (25 picomoles) was required. The same knockdown can be achieved using a 600 µL of 15 nM HIF-1α loaded DMC (10 picomoles). Only a fraction of these DMCs are functionalized to the surfaces. From SLB calibration, we know a concentration of ~13 nM yields a surface density of 3.01±0.27 femtomoles/cm^2. For an ATTO chamber the surface area is ~3 cm^2 which corresponds to ~10 femtomoles which corresponds to a ~1000-fold increase in potency.

Supplementary Figure 12: Flow Cytometry gating strategy

The following gating strategy was used in flow cytometry for gating MEF cells. A similar gating strategy was employed for HeLa cells.

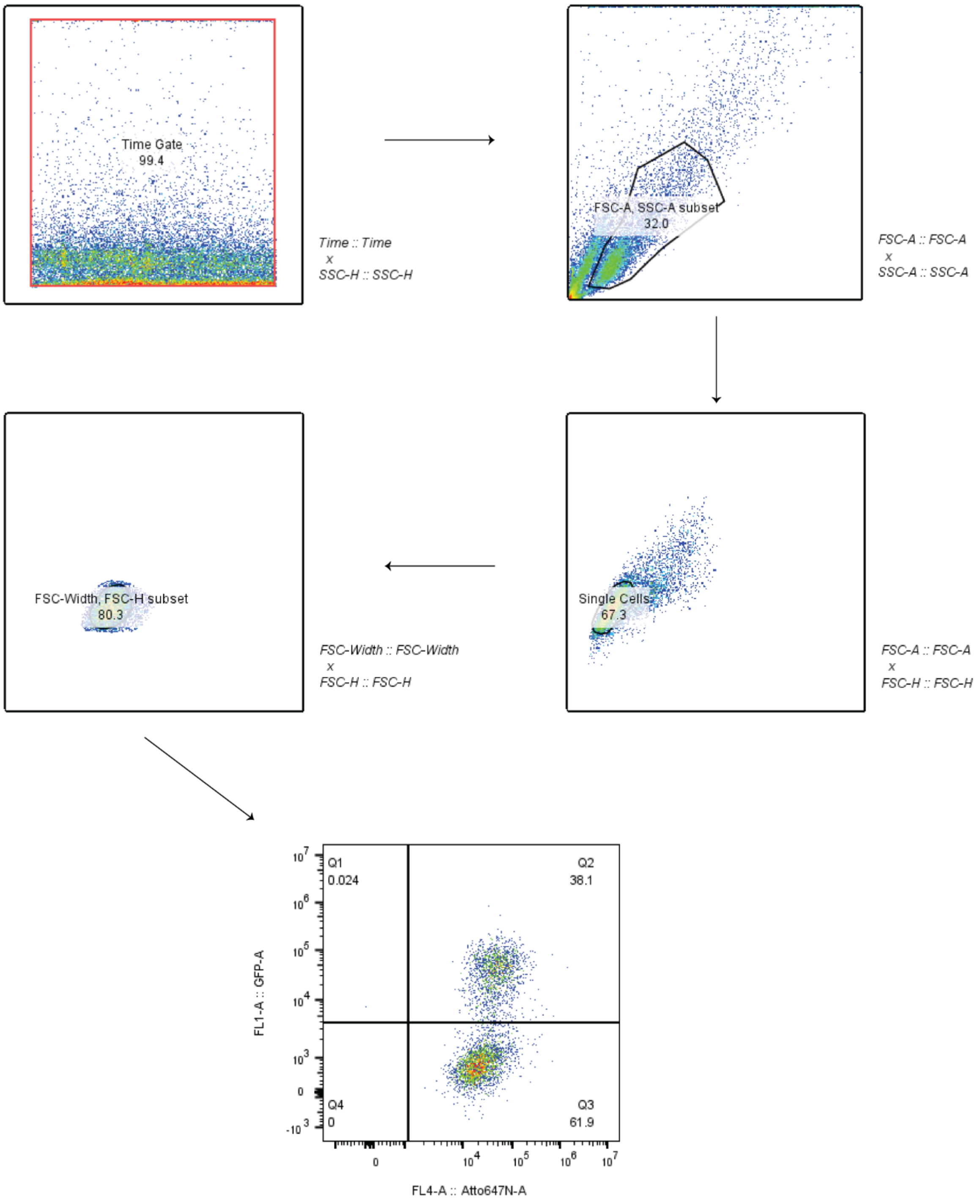

Supplementary Figure 13: Uncropped gel images

Uncropped images of the gels are shown below.

Supplementary Figure 6A

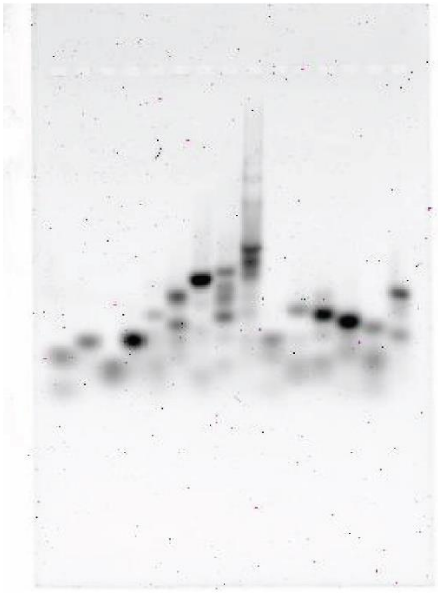

Supplementary Figure 6B

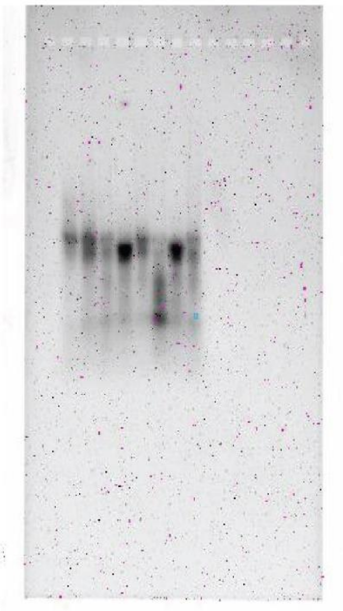

Supplementary Figure 6D

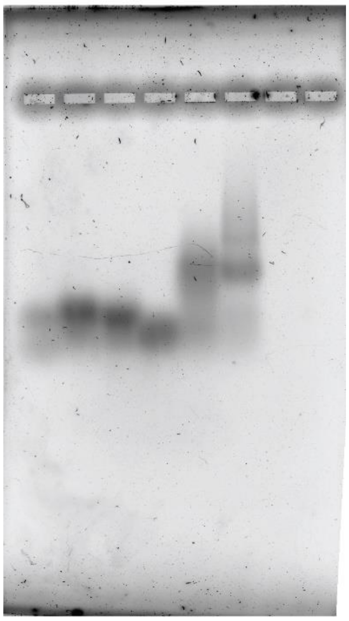

Supplementary Figure 8B

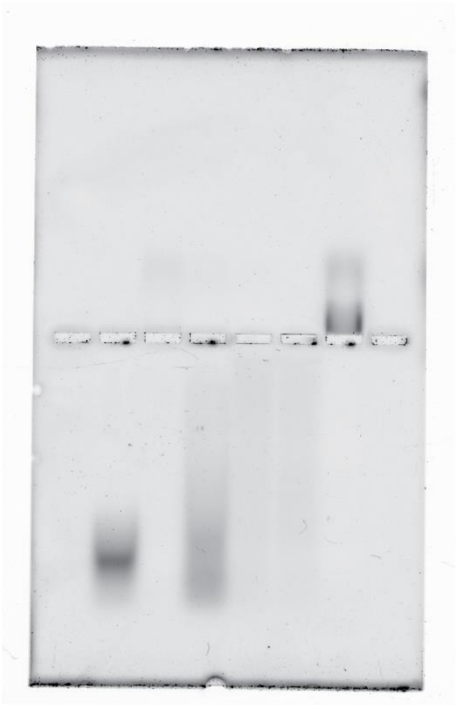

Supplementary Figure 8B

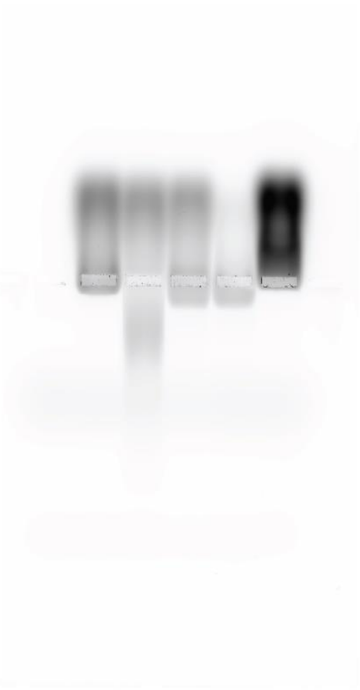

Supplementary Figure 8D

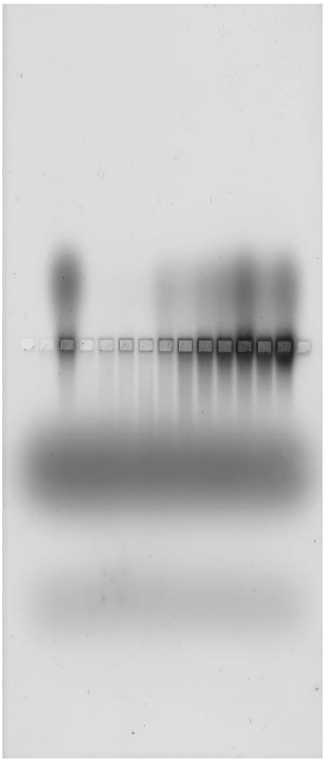

Supplementary References

1. Korin, N. *et al.* Shear-Activated Nanotherapeutics for Drug Targeting to Obstructed Blood Vessels. *Science* **337**, 738–742 (2012).
2. Stejskalová, A., Oliva, N., England, F. J. & Almquist, B. D. Biologically Inspired, Cell-Selective Release of Aptamer-Trapped Growth Factors by Traction Forces. *Adv. Mater.* **31**, 1806380 (2019).
3. Lei, K. & Tang, L. T cell force-responsive delivery of anticancer drugs using mesoporous silica microparticles. *Mater. Horiz.* **7**, 3196–3200 (2020).
4. Goodman, R. P. *et al.* Rapid Chiral Assembly of Rigid DNA Building Blocks for Molecular Nanofabrication. *Science* **310**, 1661–1665 (2005).
5. Engel, M. C. *et al.* Force-Induced Unravelling of DNA Origami. *ACS Nano* **12**, 6734–6747 (2018).
6. Harris, C. R. *et al.* Array programming with NumPy. *Nature* **585**, 357–362 (2020).
7. Virtanen, P. *et al.* SciPy 1.0: Fundamental Algorithms for Scientific Computing in Python. *Nat. Methods* **17**, 261–272 (2020).
8. McKinney, W. Data Structures for Statistical Computing in Python. in *Proceedings of the 9th Python in Science Conference* (eds. Walt, S. van der & Millman, J.) 51–56 (2010).
9. Hunter, J. D. Matplotlib: A 2D graphics environment. *Comput. Sci. Eng.* **9**, 90–95 (2007).
10. Galush, W. J., Nye, J. A. & Groves, J. T. Quantitative Fluorescence Microscopy Using Supported Lipid Bilayer Standards. *Biophys. J.* **95**, 2512–2519 (2008).
11. Vacklin, H. P., Tiberg, F. & Thomas, R. K. Formation of supported phospholipid bilayers via co-adsorption with β-d-dodecyl maltoside. *Biochim. Biophys. Acta BBA - Biomembr.* **1668**, 17–24 (2005).
