# Supplementary material for: DNA mechanocapsules for programmable piconewton responsive drug delivery
Source: Nat Commun. 2024 Jan 24;15:704. doi: 10.1038/s41467-023-44061-w (PMC10808132; doi:10.1038/s41467-023-44061-w)
Supplement: Supplementary file 3 — Description of Additional Supplementary Files [file 41467_2023_44061_MOESM3_ESM.pdf]

Description of Additional Supplementary Files

Supplementary Movie 1: Simulation of DMC 39pN in oxDNA under forces

DMC 39pN was subjected to forces along the z-axis and the trajectories were recorded.

Supplementary Movie 2: Simulation of DMC 44pN in oxDNA under forces

DMC 44pN was subjected to forces along the z-axis and the trajectories were recorded.

Supplementary Movie 3: Simulation of DMC 27pN in oxDNA under forces

DMC 27pN was subjected to forces along the z-axis and the trajectories were recorded

Supplementary Movie 4: Simulation of DMC rigid in oxDNA under forces

DMC rigid was subjected to forces along the z-axis and the trajectories were recorded
