# Supplementary material for: DNA mechanocapsules for programmable piconewton responsive drug delivery
Source: Nat Commun. 2024 Jan 24;15:704. doi: 10.1038/s41467-023-44061-w (PMC10808132; doi:10.1038/s41467-023-44061-w)
Supplement: Supplementary file 9 — Reporting Summary [file 41467_2023_44061_MOESM9_ESM.pdf]

Corresponding author(s): Khalid Salaita

Last updated by author(s): Oct 29, 2023

Reporting Summary

Nature Portfolio wishes to improve the reproducibility of the work that we publish. This form provides structure for consistency and transparency in reporting. For further information on Nature Portfolio policies, see our Editorial Policies and the Editorial Policy Checklist.

Statistics

For all statistical analyses, confirm that the following items are present in the figure legend, table legend, main text, or Methods section.

n/a	Confirmed	
☐	☒	The exact sample size (*n*) for each experimental group/condition, given as a discrete number and unit of measurement
☐	☒	A statement on whether measurements were taken from distinct samples or whether the same sample was measured repeatedly
☐	☒	The statistical test(s) used AND whether they are one- or two-sided<br>*Only common tests should be described solely by name; describe more complex techniques in the Methods section.*
☒	☐	A description of all covariates tested
☐	☒	A description of any assumptions or corrections, such as tests of normality and adjustment for multiple comparisons
☐	☒	A full description of the statistical parameters including central tendency (e.g. means) or other basic estimates (e.g. regression coefficient) AND variation (e.g. standard deviation) or associated estimates of uncertainty (e.g. confidence intervals)
☐	☒	For null hypothesis testing, the test statistic (e.g. *F*, *t*, *r*) with confidence intervals, effect sizes, degrees of freedom and *P* value noted<br>*Give P values as exact values whenever suitable.*
☒	☐	For Bayesian analysis, information on the choice of priors and Markov chain Monte Carlo settings
☒	☐	For hierarchical and complex designs, identification of the appropriate level for tests and full reporting of outcomes
☒	☐	Estimates of effect sizes (e.g. Cohen's *d*, Pearson's *r*), indicating how they were calculated

Our web collection on statistics for biologists contains articles on many of the points above.

Software and code

Policy information about availability of computer code

Data collection	NIS_elements (vS.2.1) was used for microscopy image and video recording. OpenlAB CDS chem station edition from Agilent technologies was used for HPLC data collection. CytExpert (v2.3) was used for flow data collection. LightCycler" 96 Application Software (vl.1.0.1320) was used for RT-qPCR data collection and cycle value (Cq value) estimation. BioTek plate reader with Gen 5 (v3.13.15) was used for fluorescence plate assay data collection. oxDNA simulation (v2.4 June 2019) movies were generated using oxView web-server (sulcgroup.github.io/oxdna-viewer/).
Data analysis	Code used for oxDNA analysis is available on github.com/Arventh/DNA_MechanoCapsules. Fiji was used for image analysis (fiji.sc). FlowJo (vl0.8.1) was used for flow data analysis.

For manuscripts utilizing custom algorithms or software that are central to the research but not yet described in published literature, software must be made available to editors and reviewers. We strongly encourage code deposition in a community repository (e.g. GitHub). See the Nature Portfolio guidelines for submitting code & software for further information.

Data

Policy information about availability of data

All manuscripts must include a data availability statement. This statement should provide the following information, where applicable:

- Accession codes, unique identifiers, or web links for publicly available datasets
- A description of any restrictions on data availability
- For clinical datasets or third party data, please ensure that the statement adheres to our policy

Research data including oxDNA input and code used for analysis is available at zenodo.org/uploads/10052232

Research involving human participants, their data, or biological material

Policy information about studies with human participants or human data. See also policy information about sex, gender (identity/presentation), and sexual orientation and race, ethnicity and racism.

Reporting on sex and gender	N/A
Reporting on race, ethnicity, or other socially relevant groupings	N/A
Population characteristics	N/A
Recruitment	N/A
Ethics oversight	N/A

Note that full information on the approval of the study protocol must also be provided in the manuscript.

Field-specific reporting

Please select the one below that is the best fit for your research. If you are not sure, read the appropriate sections before making your selection.

☒ Life sciences ☐ Behavioural & social sciences ☐ Ecological, evolutionary & environmental sciences

For a reference copy of the document with all sections, see nature.com/documents/nr-reporting-summary-flat.pdf

Life sciences study design

All studies must disclose on these points even when the disclosure is negative.

Sample size	No sample size calculation was performed. All experiments were repeated to have 3 - 4 biological replicates. For cell imaging, 10-15 images were captured in a given biological replicate. RT-qPCR were performed with 2-3 technical replicates for a biological replicate (same batch of cells on 2-3 independent surfaces) as well 2 technical replicates for RT-qPCR run of RNA from a given surface to minimize pipetting errors. For Flow experiments, about 10,000 cells were recorded before gating. These sample sizes were sufficient to represent the experimental results.
Data exclusions	Images of surfaces (with or without cells) were only used for analyses if the image was clear, in-focus and free from large imperfections. Few cells with areas larger than the image frame are were excluded to avoid underestimation of cell spread area. In RT-qPCR experiments, replicates were excluded in the following 1) surfaces breaking during the experiment (6-24 hours) and leaked cell media 2) infection of the cells on surface resulting in cloudy media and/or dead cells. 3) impure/insufficient RNA extraction from the cells on the surface. 4) technical replicates with anomalous 18S Cq values run from the same sample. In all RT-qPCR runs, the controls were ran alongside the treated samples as it is required for normalization and in cases where all technical replicates of the control sample were lost (due to reasons described above) the entire experiment is discarded.
Replication	All experiments involving cells and/or DNA functionalized surfaces were repeated at least 3 times with different batches of surfaces, cells on different days to ensure reproducibility. In RT-qPCR experiments, each biological replicate was done with 2-3 technical replicates (2-3 surfaces with cells from a given batch on a given day) to improve the success rate of high quality RNA extraction from the cells. The extracted RNA was analyzed using RT-qRCR as 2 technical replicates to account for pipetting errors. All attempts at replication for RT qPCR experiments were successful.
Randomization	Randomization was not performed since it was not necessary to study the parameters or perform the techniques described.
Blinding	No group allocation was performed hence no blinding was used in the study.

Reporting for specific materials, systems and methods

We require information from authors about some types of materials, experimental systems and methods used in many studies. Here, indicate whether each material, system or method listed is relevant to your study. If you are not sure if a list item applies to your research, read the appropriate section before selecting a response.

Materials & experimental systems

n/a	Involved in the study
☒	☐ Antibodies
☐	☒ Eukaryotic cell lines
☒	☐ Palaeontology and archaeology
☒	☐ Animals and other organisms
☒	☐ Clinical data
☒	☐ Dual use research of concern
☒	☐ Plants

Methods

n/a	Involved in the study
☒	☐ ChIP-seq
☐	☒ Flow cytometry
☒	☐ MRI-based neuroimaging

Eukaryotic cell lines

Policy information about cell lines and Sex and Gender in Research

Cell line source(s)	Mouse embryonic fibroblasts (vinculin null, 3T3 paxillin-GFP and vinculin-GFP) were a gift of Prof. Andres Garcia lab (other). NIH3T3 cells were obtained from ATCC (commercial). Hela, MCF-7, MCF-l0a, and MDA-MB-231 were all gifts from labs at Winship Cancer Institute, Emory University (other).
Authentication	Cell lines have not been authenticated.
Mycoplasma contamination	Cell lines were not checked for mycoplasma contamination.
Commonly misidentified lines (See ICLAC register)	No misidentified cell lines were used.

Plants

Seed stocks	N/A
Novel plant genotypes	N/A
Authentication	N/A

Flow Cytometry

Plots

Confirm that:

☒ The axis labels state the marker and fluorochrome used (e.g. CD4-FITC).

☒ The axis scales are clearly visible. Include numbers along axes only for bottom left plot of group (a 'group' is an analysis of identical markers).

☒ All plots are contour plots with outliers or pseudocolor plots.

☒ A numerical value for number of cells or percentage (with statistics) is provided.

Methodology

Sample preparation	Cells were detached using trypsin or EDTA and was washed and injected into FlowCytometer. See Methods section for further details.
Instrument	CytoFLEX V0-B3-Rlwith 488nm and 638nm lasers
Software	CytExpert v2.3, FlowJo vl0, Graph Pad Prism
Cell population abundance	Shown in Supplementary figure 511. Briefly, median was calculated from samples with at least 10000 events before any gating.

Gating strategy

Shown in Supplementary figure 11

☒ Tick this box to confirm that a figure exemplifying the gating strategy is provided in the Supplementary Information.
